# BioTAGME: A Comprehensive Platform for Biological Knowledge Network Analysis

**DOI:** 10.3389/fgene.2022.855739

**Published:** 2022-04-28

**Authors:** Antonio Di Maria, Salvatore Alaimo, Lorenzo Bellomo, Fabrizio Billeci, Paolo Ferragina, Alfredo Ferro, Alfredo Pulvirenti

**Affiliations:** ^1^ Department of Clinical and Experimental Medicine, University of Catania, Catania, Italy; ^2^ Scuola Normale Superiore, Pisa, Italy; ^3^ Department of Maths and Computer Science, University of Catania, Catania, Italy; ^4^ Department of Computer Science, University of Pisa, Pisa, Italy

**Keywords:** knowledge graph, text mining, annotation tools, TAGME, wikipedia, DT-hybrid

## Abstract

The inference of novel knowledge and new hypotheses from the current literature analysis is crucial in making new scientific discoveries. In bio-medicine, given the enormous amount of literature and knowledge bases available, the automatic gain of knowledge concerning relationships among biological elements, in the form of semantically related terms (or entities), is rising novel research challenges and corresponding applications. In this regard, we propose BioTAGME, a system that combines an entity-annotation framework based on Wikipedia corpus (i.e., TAGME tool) with a network-based inference methodology (i.e., DT-Hybrid). This integration aims to create an extensive Knowledge Graph modeling relations among biological terms and phrases extracted from titles and abstracts of papers available in PubMed. The framework consists of a back-end and a front-end. The back-end is entirely implemented in Scala and runs on top of a Spark cluster that distributes the computing effort among several machines. The front-end is released through the Laravel framework, connected with the Neo4j graph database to store the knowledge graph.

## 1 Introduction

The increasing amount of scientific literature is raising new challenges for scientists. For example, identifying the proper set of articles dealing with a specific topic could be a not straightforward task. Thus, the possibility of missing essential references and relevant research is high nowadays. In particular, in research areas such as Biology or Bio-Medicine, thanks to fast-track publication journals, the number of published papers increases significantly fast, thus making it very difficult for scientists to keep track of literature evolution.

Furthermore, network analysis has become a key enabling technology to help the understanding of life mechanisms, living organisms and, in general, and uncover the underlying fundamental biological processes. Examples of applications include 1) analyzing disease networks for identifying disease-causing genes and pathways Barabási et al. (2010); 2) discovering the functional interdependence among molecular mechanisms through functional network querying (Xiaoke and Lin (2012)); 3) deriving network-based inferences for drug repurposing (Himmelstein et al. (2017)).

The large number of publicly available ontologies, which hold entities and their relations (Lambrix et al. (2007)), and the repositories of open-access articles such as PubMed Central (Beck (2010)), arXiv, and bioarXiv, are driving the academic community to rely on text mining tools and machine learning algorithms for extracting *semantic knowledge* from documents such as understanding how proteins interact each other, which gene mutations are involved in a disease, etc. In this context, the Biological Expression Language (BEL) (Hoyt et al. (2018)) or the Resource Description Framework (RDF) (McBride (2004)) are widely employed to represent this *knowledge* as triplets having the following structure: < subject, predicate, object > . The subject and the object represent biological elements, whereas the predicate represents a (logical or physical) relationship.

Since the implementation of biological text mining methodologies requires skills in natural language processing (NLP) that usually end-users do not have, several tools have been made available to scientists: 1) PubAnnotation (Kim et al. (2019)) is based on the “Agile text mining” concept, and it is a public resource for sharing annotated biomedical texts; 2) PubTator (PTC, Wei et al. (2019)) is a web service for viewing and retrieving bio-concept annotations (for genes/proteins, genetic variants, diseases, chemicals, species, and cell lines) from all PubMed abstracts and more than three million PubMed full-texts. These annotations are downloadable in multiple formats (XML, JSON, and tab-delimited) via the online interface, a RESTful web service, and bulk FTP. PTC is synchronized with PubMed and PubMed Central, adding new articles daily.

The literature also offers many frameworks for building functional networks. **STRING** (Szklarczyk et al. (2016)) is a database that collects known and predicted functional protein-protein associations for many organisms. Each protein-protein association is given a score (between zero and one) which summarizes the biological reliability of the interaction, its specificity, and the supporting evidence. Another significant contribution of these interactions is the so-called “interolog” transfer, based on the observation that orthologs of interacting proteins in one organism are often also interacting in another organism. The STRING resource is available online^1^. **Hetionet** (Himmelstein et al. (2017) is a heterogeneous network of biomedical knowledge constructed over genes, diseases, and compounds, extracted from the processing of a collection of 29 publicly available databases and millions of publications. It was created as part of Project Rephetio to predict new uses for existing drugs. In the last few years, it has been modified for working over a wider variety of purposes: such as drug repurposing and prioritizing disease-associated Genes. Hetionet is available at^2^ **Reactome** (Croft et al. (2010) is a peer-reviewed knowledge base of biomolecular pathways that contains a detailed representation of cellular processes interconnecting terms to form a graph modeling biological knowledge. Reactome adopts Neo4j as a graph database to improve the graph traversal performance and knowledge discovery. Reactome is also available online^3^. **SemRep** (Rindflesch and Fiszman (2003)) is an NLP advanced information management application, which extracts relationships from biomedical sentences in PubMed titles and abstracts by mapping textual content to an ontology representing its meaning. To establish the binding relation, SemRep relies on internal rules (called “indicator rules”), which map syntactic elements, such as verbs, prepositions, and nominalization, to predicates in the Semantic Network. It is available at^4^ **Kindred** (Lever and Jones (2017)) is a Python package built on top of the Stanford CoreNLP framework and the scikit-learn library. It performs relation extraction in biomedical texts, where relation candidates are created by finding every possible pair of entities within each sentence. Next, it exploits an SVM classifier to rank and select the most promising candidates. In **NetME** (Muscolino et al. (2022)), authors propose a tool that allows to query PUBMED and build knowledge networks synthesizing the concepts described through the selected papers. In the context of clinical Text Analysis and Knowledge Extraction, **cTAKES** (Savova et al. (2010)) is a system for information extraction from electronic medical record free-text. The pipeline comprises several modules, such as sentence boundary detector, tokenizer, normalizer, part-of-speech tagger, Shallow parser, and named entity recognizer. Other relevant work include **CKG** (Santos et al. (2022)). CkG is an open-source knowledge-graph platform, which includes 20 million nodes and 220 million relationships that represent relevant experimental data, public databases and literature. CKG incorporates statistical and machine learning algorithms to accelerate the analysis and interpretation of common proteomics workflows.

This paper introduces BioTAGME, a knowledge graph inferred from more than 33 million titles and abstracts in the PubMed database (Williamson and Minter (2019)), and downloadable as XML files via third-party applications.

BioTAGME uses two well-known tools to generate the Knowledge Graph. First, entities are extracted from each abstract using the TAGME annotation system (Ferragina and Scaiella (2010)). TAGME is a tool that analyzes short texts and extracts entities related to its content. It makes use of Wikipedia to perform the annotation. All the entities extracted from the abstracts are treated as nodes of the knowledge graph. Next, the DT-Hybrid (Alaimo et al. (2013)) recommendation system is applied to predict possible relationships among entities coming from different abstracts. These relationships form the edges of the knowledge graph. Finally, such predicted relationships are enriched with those from publicly available databases (the complete list is provided in Section 2) to generate a comprehensive Knowledge Graph, stored in the Neo4j database and made available to users via our web app. Such a knowledge graph consists of more than 161 thousand nodes and 40 million edges. Moreover, there are three different types of edges: 1) Literature edge: indicates a piece of biological evidence resulting from laboratory experiments, biological and biophysical processes; 2) STRING edge: represents STRING predicted protein-protein associations; finally 3) BioTAGME edge: are edges predicted by the combination of TAGME relatedness and BioTAGME one. Both BioTAGME edges and STRING ones are marked with the corresponding score value to indicate the interaction’s likelihood. Biotagme is available at: https://biotagme.eu/ ^5^

The paper is organized as follows. In the Section 2, we introduce the back-end of our tool. Next, we introduce the web app to browse and query the system. Moreover, we show a BSG-Diseases network that reports literature evidence and BioTAGME prediction. Finally, in section conclusions, we explain future work about our tool.

## 2 Materials and Methods

**FIGURE 1:**
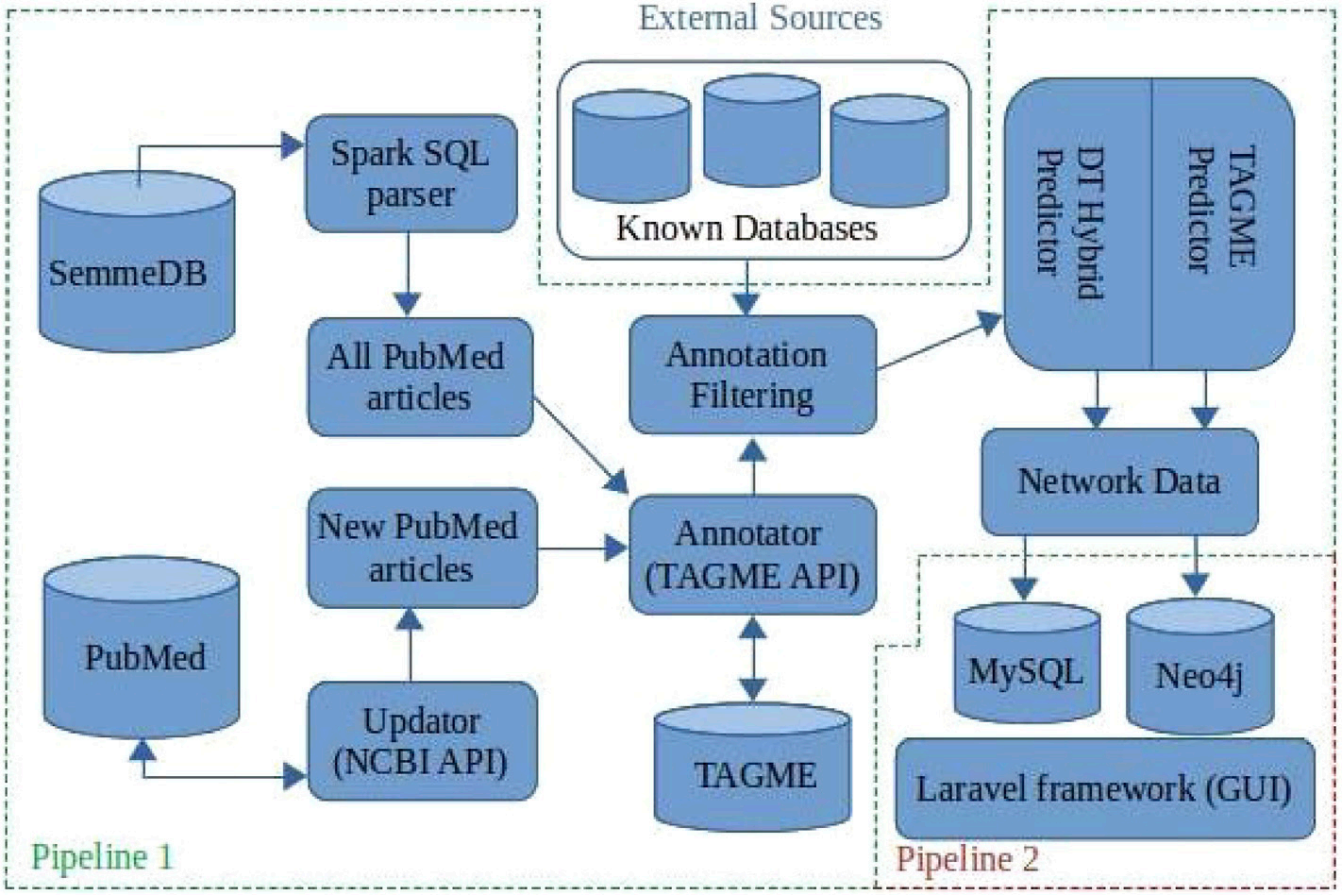
BioTAGME pipelines: The first one pipeline is the core of the project. It transforms the whole set of PubMed abstracts within nodes and edges of the knowledge graph. It has been implemented in Scala e Spark. The seconds pipeline, allows a user to extract information from the graph. It has been implemented using Laravel and Reactstrap.

BioTAGME is a framework backed by two different pipelines (Figure 1) for building a biological knowledge graph from PubMed documents’ titles and abstracts. It integrates two different learning algorithms, DT-Hybrid (Alaimo et al. (2013)) and TagME (Ferragina and Scaiella (2010)).

The first pipeline is built on top of the Apache SPARK analytic engine and Hadoop Distributed File System (HDFS). This implementation guarantees large-scale data processing through cluster managers (Apache Meson, YARN, Stand Alone, and Kubernetes). The pipeline collects results into DataFrames (Apache-Spark (2016)) the data coming from several freely available online databases as shown in Table 1. In addition, the complete set of PubMed titles and abstracts in order to build a life science knowledge graph using the Spark SQL language. DataFrame and SQL language provide a common way to access various data files, including Hive, Avro, Parquet, CSV, TSV, and JSON.

**TABLE 1:** Ontologies.

Source name	Citation	Data type
DisGeNET	Piñero et al. (2019)	human gene-disease association
DiseaseOntology (DO)	Schriml et al. (2018)	human disease
DiseaseEnhancer	Zhang et al. (2017)	human disease-associated enhancer
DrugBank	Wishart et al. (2007)	drug and drug target
PharmGKB	Thorn et al. (2013)	human-genetic variation on drug resp
HGNC	Daugherty et al. (2012)	human gene
ENSEMBL	Birney et al. (2004)	vertebrates genomic information
LNCipedia	Volders et al. (2012)	human long non-coding RNAs
miRcode	Jeggari et al. (2012)	human microRNA-target predictions
miRBase	Kozomara et al. (2018)	microRNA sequences
miRTarBase	Huang et al. (2019)	microRNA-target interactions
miRCancer	Xie et al. (2013)	microRNA expression profile in cancer
Reactome	Fabregat et al. (2017)	pathway
PathBank	Wishart et al. (2019)	pathway
UniProt	The UniProt Consortium (2016)	protein sequence
STRING	Szklarczyk et al. (2018)	protein–protein interaction
BRENDA	Chang et al. (2020)	enzyme

The major functionalities provided by the first pipeline are 1) Download and import, 2) SQL to JSON parser, 3) Integrating databases, 4) Annotation, 5) Prediction, 6) Network generation, and 7) Updating.

The second pipeline is built on top of the Laravel framework and consists of the following components: 1) MySQL for storing names, aliases, BioTAGME IDs, and Wikipedia pages IDs; 2) Neo4j for storing the knowledge graph, and allow querying the network (i.e., compute the shortest path between two user-specified biological entities (nodes)); 3) the User Interface (GUI), based on Laravel and React, used for wrapping the Neo4j queries and making them more accessible and more intuitive. Queries can be: 1) Search on the graph; 2) Shortest path. (Detailed information are in Section 2.2).

Data processing is done in PHP and bash to achieve high performance. In addition, all the GUI modules have been realized in react-native.

### 2.1 Pipeline One: Data Loader and Network Synthesis

This section describes all components and functionalities of the first pipeline underling BioTAGME.

#### 2.1.1 Download and Import Module

This module allows importing the external databases into Hadoop Distributed File System (HDFS) through a custom bash script, which consists of three main sections:

• PubMed section: it downloads titles and abstracts of PubMed articles through SemmedDB SENTENCE table (Kilicoglu et al. (2012)). Such table contains all the sentences related to the articles’ title and abstract in PubMed.
• Literature databases section: it downloads the external databases which are used for i) filtering of noisy annotation entities caused by disambiguation and high generality of the Wikipedia corpus; ii) building literature edges, a biological evidence resulting from laboratory experiments, biological and biophysical processes. These edges allow us to evaluate the quality of BioTAGME prediction. Note that some databases, such as DrugBank (Wishart et al. (2007)), PharmGKB (Thorn et al. (2013)), Brenda (Chang et al. (2020)), require free registration or authorization to be downloaded. Therefore, such a procedure is left to the user.
• The import section transfers the downloaded databases from the local file system to the Hadoop FileSystem (HDFS).

#### 2.1.2 SQL to JSON Parser Module

Although SemmedDB guarantees faster downloads than NCBI Entrez APIs, it has two main issues: the 1) title and abstract of each PMID (Document identifier in PubMed) are divided into sentences, and 2) the SENTENCE table is in a SQL format, which is not natively supported by the Spark engine.

To solve these issues, we implemented a new Spark module, named SQL2Json parser, that extracts headers, and every data row from a table by applying Spark SQL Window methodology. Each row is then aggregated to form the complete title and abstract through Spark built-in collect_list, concat_ws, and group-by functions. Finally, the parsed data is converted into JSON format and stored within the Hadoop FileSystem.

#### 2.1.3 External Databases Integration Module

As previously mentioned, several databases are integrated into our pipeline. However, there are a few issues to consider: 1) Different databases often use different words to describe the same entity (synonyms). For example, DisGenNET uses “Colorectal cancer, hereditary nonpolyposis, type 1”, while DiseaseOntology (DO) uses “Lynch syndrome 1” to refer to the same disease. 2) Equivalent attributes have different names in different databases. For example, a database might use the attribute name “mirna_nr”, while another database might use “id”. 3) Different databases might use different files formats, such as JSON, XML, TXT, CSV, TAB, OBO, GTF, FASTQ, and SQL, etc.

We implemented an integration module that executes the following tasks to tackle such issues. First, all databases are loaded into Spark DataFrames. We use the built-in Spark functions for CSV (read.csv), Tab-delimited and TXT (read.txt), and JSON (read.json) files. To import OBO, GFT, SQL, and FASTQ files, we implemented custom spark modules that convert such formats into DataFrames. The Databricks Spark-XML (Databricks (2021)) library is used for XML files. Then, each DataFrame is processed and subjected to a schema redefinition by using external databases metadata, synonyms list, and references (toward other external databases) list to harmonize the contents of the different data sources. This module is a fundamental intermediate layer that transforms all external databases into new ones having the same schema, attributes, format, and nomenclature.

#### 2.1.4 Annotation Module

This module transforms documents’ titles and abstracts into a list of annotation entities. Thus, for each document “t_*i*_”, a tuple (TI_AB, TAGME parameters map)_*i*_ is generated and sent to the TAGME API through an HTTP POST request. We use TI_AB to represent the union of Document_*i*_ Title and Abstract.

TAGME removes all stop-words and punctuation symbols from the TI_AB text at first. Then, a list of “annotation entities” is extracted and returned in response to the request, where each entity can be one or more words. Each annotation entity contains entity text, Wikipedia page title, Wikipedia page categories, and Wikipedia page ID. Each entity will be a node of the knowledge graph.

TAGME annotations are not entirely accurate. The authors provide an estimate *F*_1_ measure of 0.78, where *F*_1_ is the harmonic mean between the precision and the recall of the annotation process. However, this does not considers any improvement due to 1) more up-to-date Wikipedia dumps and 2) pages filtering to obtain only Wikipedia pages relevant to the Biological field. Indeed, we properly pruned the Wikipedia network using the main biological categories^6^ to 1) perform annotation only on Biological entities, and 2) mitigate the disambiguation problem.

Finally, the documents with their annotation entities are sent to the prediction module to generate the relationships.

#### 2.1.5 Prediction Module

Our methodology aims to predict a potential relationship between *i*-th entity and *j*-th entity based on the BioTAGME score value (*BioTG*_*i*,*j*_). This score is defined as the product between the DT-Hybrid score *s*_*i*,*j*_ (Alaimo et al. (2013)) and the TAGME relatedness one *r*_*i*,*j*_ (Ferragina and Scaiella (2010)). The higher is the score value, the higher is the meaningfulness of the predicted relationship.

The domain tuned-hybrid (DT-Hybrid) tool (Alaimo et al. (2013)) defines a recommendation method based on a bipartite network projection technique that implements the concept of resources transfer within the network to predict the robustness of the relationship between a pair of entities.

The DT-Hybrid score is computed by using a DT-Hybrid version running on Spark; the TAGME relatedness is computed through the online TAGME service available at^7^. The relatedness value is in the range [0,1] and expresses how much two entities are semantically related within the Wikipedia corpus. The value zero means no relationships between them; the value one means equivalence between two entities.

The output of this step is a set of relations between entities. These relations are then integrated during the network-construction phase with others coming from the external databases.

#### 2.1.6 Network Construction

As soon as the documents have been annotated and the prediction procedure has been completed, the last step of the pipeline is to build the Knowledge Graph containing logical or physical relationships among biological elements. Physical relationships represent the real connection between biological entities. Instead, the logical one represents the effect that a biological entity (i.e., Drug) could have on another one (i.e., Disease or Gene).

For every *Entity*_*i*_–*Entity*_*j*_ association obtained during the prediction procedure, our system creates three different edges types:

• Literature: indicates an interaction derived from a publication, describing a biological evidence resulting from laboratory experiments, biological, and biophysical processes, etc.
• STRING: represents the predicted protein-protein associations stored in the STRING database. We report this information because our system integrates STRING *Homo sapiens* protein-protein interactions.
• BioTAGME: the edges predicted by our tool.

Both BioTAGME edges and STRING edges are marked with the corresponding score value to indicate the interaction’s likelihood. More information about the plotting of the network, motif search, and shortest path computations are reported in the following Section 2.2.

We publicly release our network on Zenodo. The link is provided in the Supplementary Data section. Data is fully compliant with *FAIR* principles (Wilkinson et al. (2016)).

#### Supplemental Data

The networks data (nodes, edges, and other files) are available at: https://doi.org/10.5281/zenodo.6325345360.

The pipeline one code is available at: https://github.com/Anto188bas/biotagme pipeline.git361

The pipeline two code is available at: https://github.com/Anto188bas/biotagme laravel.git362

The docker-compose.yml file is available at: https://github.com/Anto188bas/biotagme docker.git363

#### 2.1.7 Updating Procedure

BioTAGME pipeline annotates Pubmed documents’ titles and abstracts to predict the relationships among their corresponding biological entities. A periodical update is needed since many new documents are submitted daily to the Pubmed database.

Our pipeline carries out the following steps to achieve this purpose. First, it downloads all the PMIDs (Documents’ identifier in PubMed) within an established data range [mindate, maxdate] through an NCBI esearch POST request. “Mindate” usually refers to the last updating date; whereas “maxdate” is usually set to the actual date.

Once the PMIDs list has been obtained, the updating module downloads the title and abstract of these PMIDs using the NCBI efetch API. For performance reasons, the PMIDs list is partitioned into chunks of proper size, and then several chunk-based NCBI efetch post requests are generated and sent to the Pubmed server to obtain the required data. NCBI does not impose a maximum on the number of requests to be submitted, especially when a POST request is used. However, we suggest keeping this value under 10,000 to reduce the computational burden of our job.

Once the documents’ titles and abstracts have been downloaded, the annotation, prediction, and network construction procedures are executed to update the Knowledge Graph’s edges and nodes.

The update procedure is incremental. It does not require the entire PubMed abstracts corpus. It runs on a subset of abstracts within a date range ([start_date, end_date]), and then generate a knowledge graph only on those abstracts. Therefore, this procedure could be used to produce a temporal knowledge graphs over a certain topic of interest.

### 2.2 Pipeline Two: Network Deployment and Query Interface

**FIGURE 2:**
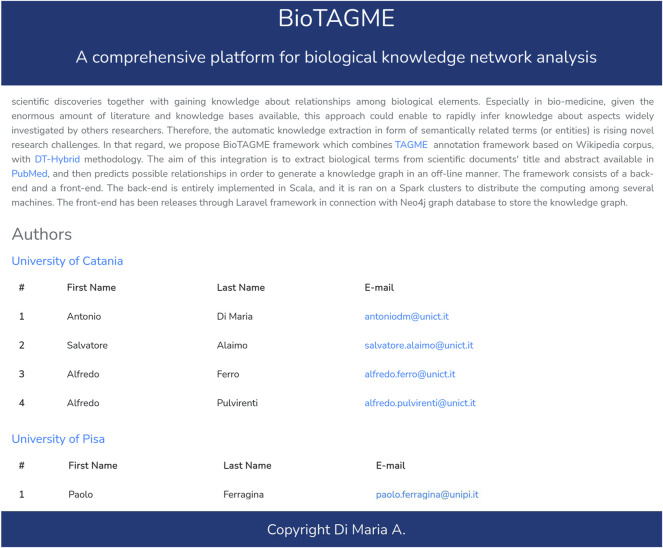
BioTAGME homepage.

The second pipeline has been implemented for importing the Knowledge Graph into the Neo4j database and querying the network to get the neighborhood of a biological element or compute the shortest path between two nodes. The interface module for network querying is crucial to exploit such graphs and infer putative novel biological knowledge. This pipeline employs the Laravel model-view-controller and the React Native framework to implement the back-end and web-pages components. In this section, we will describe such modules (Figure 2).

#### 2.2.1 Network Import Module

**FIGURE 3:**
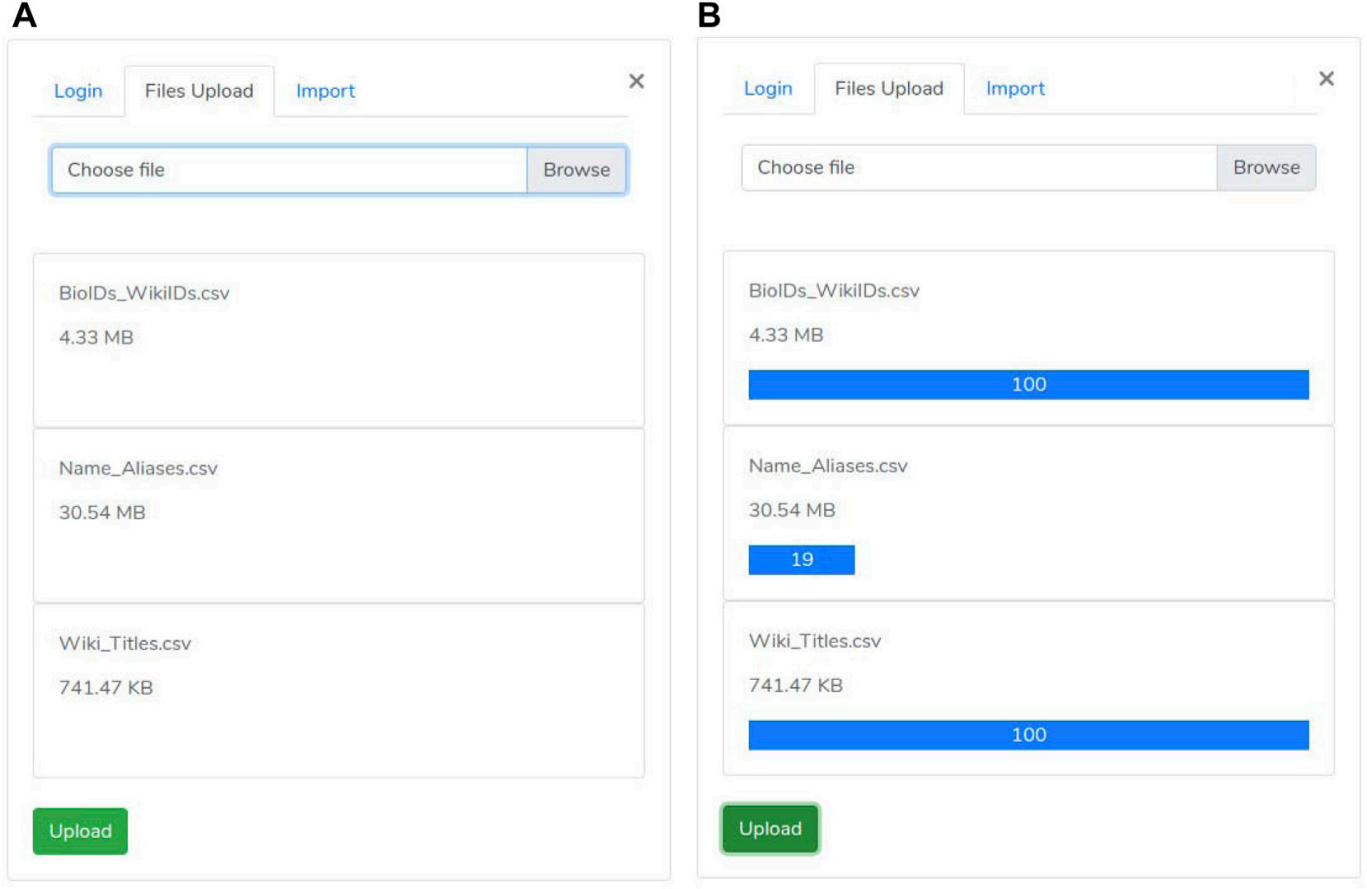
Upload Panel: Such a panel allows a manager user of the site to load the graph and metadata. **(A)** File upload panel; **(B)** File upload panel with progress bar displaing the upload status.

**FIGURE 4:**
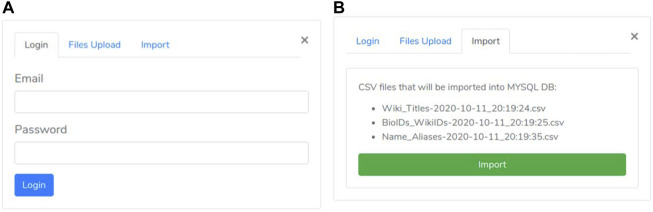
Authentication and Import panel. **(A)** Authentication panel; **(B)** Import panel.

A user may access the upload section through the “biological element search” panel by clicking on the “network files upload” link. Such section includes three consecutive phases:

• the first one is the “authentication phase” ensuring that only authorized users may execute the import procedure (Figure 4A).
• then, the “files selection phase” is enabled (Figure 3). During this phase, the user selects both “nodes.csv” and “edges.csv” files containing the network components and the “Name_Aliases.csv” file about biological elements aliases. Since the size of the files is large (GB), our system uses the “Pion” library (Pion (2021)) to split the file into small chunks (client-side) and re-assemble them as soon as these are correctly received (server-side).
• As all files are successfully received, the “import phase” is enabled. It shows a summary (Figure 4B) of the uploaded files to check for file selection mistakes. If everything is correct, the user can trigger network loading on Neo4j by clicking the import button.

#### 2.2.2 Searching Module

**FIGURE 5:**
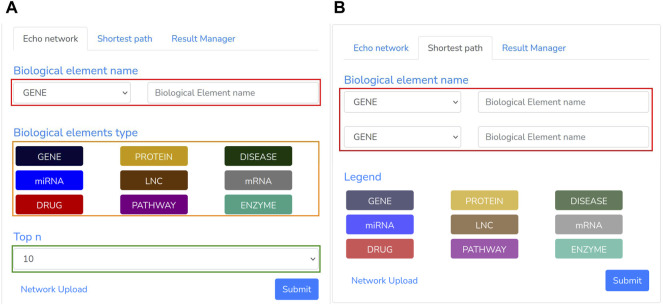
Echo Network and Shortest path panel: The first **(A)** is used to extract the neighborhood of a given node and type. **(B)** The second one, instead, returns the shortest path among two specified biological entities.

**FIGURE 6:**
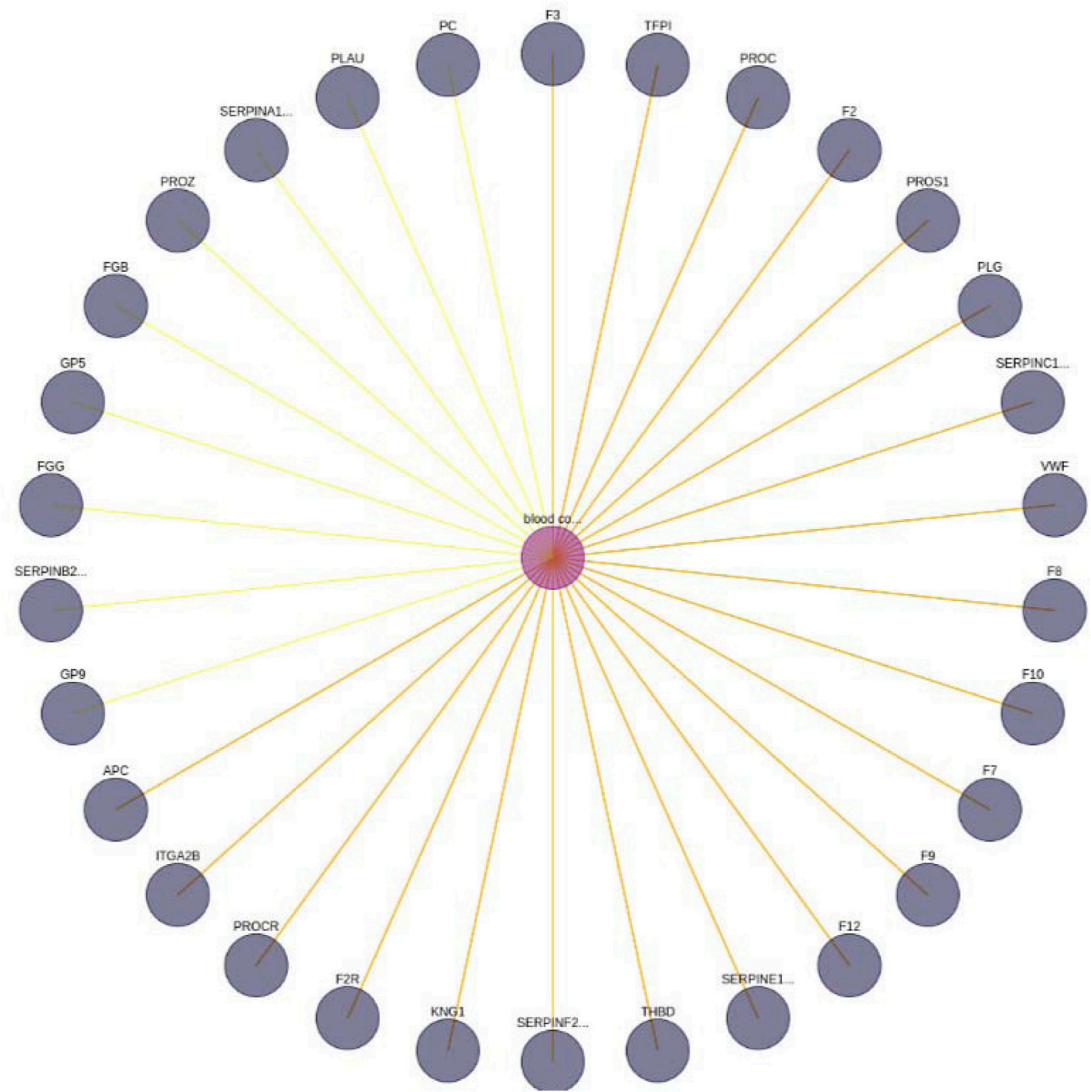
Blood coagulation—gene interaction network. A limit of 30 has been set. In addition, the yellow edges represent a set of BioTagME unpredicted edges (extracted by external databases). Instead, the orange ones (yellow + red) are edges both predicted (by BioTagME) and extracted from the external databases.

Once the network has been imported, a user may execute several queries through our “GUI”, composed of the following panels: Searching panel (Figure 5) and Graph panel (Figure 6).

The Searching Panel is used for setting the query parameters based on the selected menu: 1) Echo network or 2) shortest path.

• When the Echo Network option is selected, a user may search the Echo Network of a biological entity “be_*i*_”. Therefore, he should provide the type and name of the biological entity to be analyzed (Figure 5A, red rectangle) and the type of the other entities (Figure 5A, orange rectangle) to include within the echo network. To avoid building a large graph, a maximum number of entities has to be supplied (ranging from 10 to 200 nodes) through the “Top n” section (Figure 5A, green rectangle). Once all the required parameters have been filled, the search process can be triggered by clicking the Submit button. This process transforms the specified parameters in a “Cypher query”^8^ that looks for the “Top n” nodes having one or more links from/to “be_*i*_”.
• When the Shortest Panel option is selected (Figure 5B), a user looks for the shortest path between two biological entities. First, the user specifies the type and name of the source “*el*_*src*” and destination “*el*_*dst*” entities (Figure 5B, red rectangle), and then BioTAGME transforms all these parameters into a proper “Cypher query” which is mainly based on a Neo4j shortest path computation.

**FIGURE 7:**
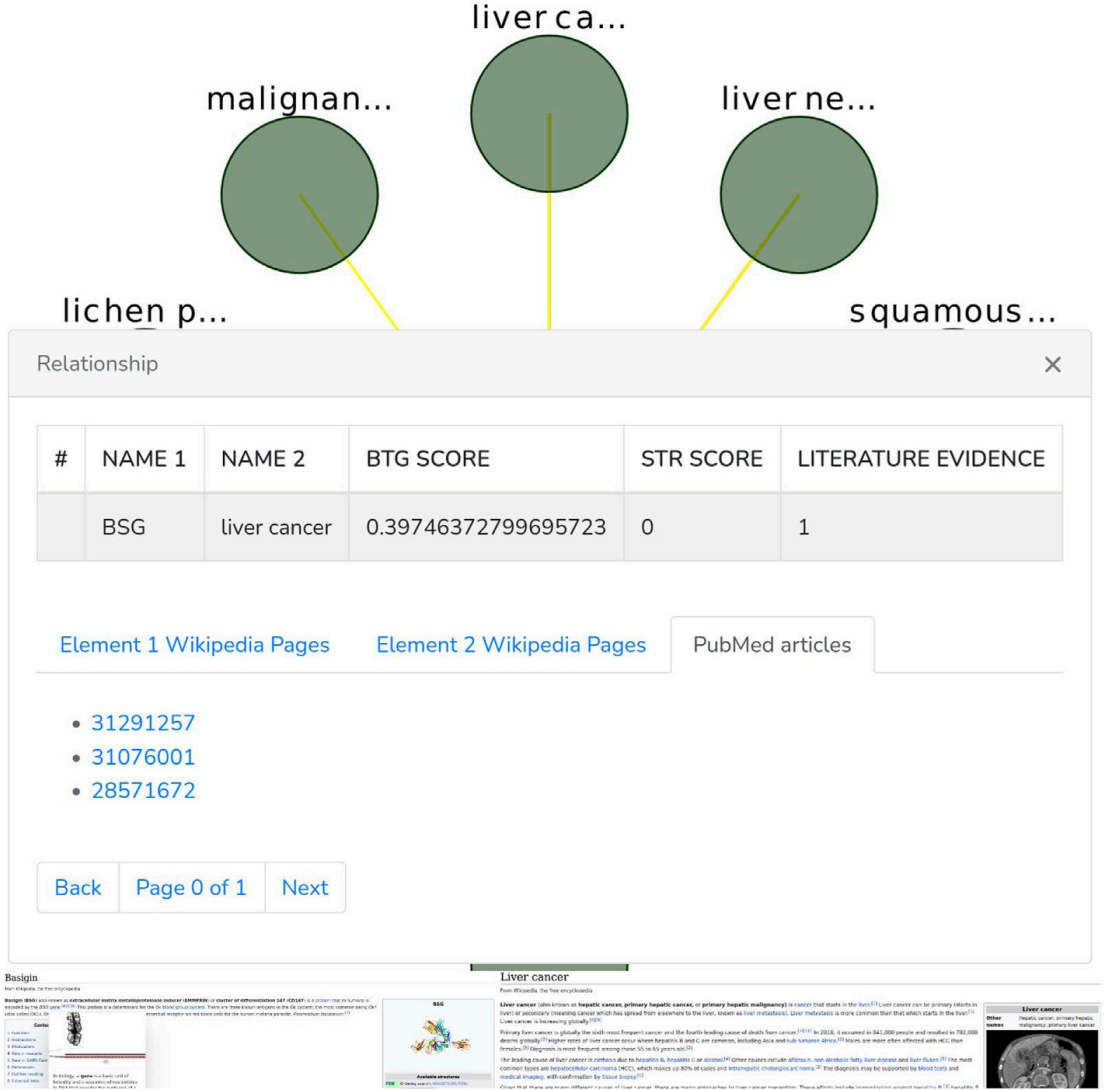
Relationship window.

The Graph phanel is used to plot [by using the CytoscapeJS library (Franz et al. (2015))] the sub-graph (Figure 6) corresponding to a user-submitted query. The edges of such sub-graph are interactive. Thus, if a user clicks on them, then a relationship window (Figure 7) containing the following data is shown:

• A table containing the name of the source and destination nodes as well as the BioTagME and STRING scores. In addition, the last column of the table also reports the literature evidence (1 if the relationship is reported in at least one of the literature databases, 0 otherwise).
• A navigation panel with three different options. The first two (Element 1 Wikipedia Pages and Element 2 Wikipedia Pages) show several links among Wikipedia pages and source or destination nodes, respectively. The last one (PubMed articles) shows all the links to PubMed articles containing the selected relationship.

## 3 Experimental Analysis

We analyzed the the reliability of BioTagMe on two case studies. The first one aims at determining preduction quality by evaluating our ability to extract “Basigin” relationships. The results were compared with STRING (Szklarczyk et al. (2018)). The second case study focuses on the construction of a “blood coagulation” network. Such a network is then compared against a literature one (generated by the links among the external databases employed in BioTagME, Table 1).

### 3.1 Case study 1

Many tools and computational models (Alaimo et al. (2020)) rely on existing network databases, such as KEGG (Kanehisa and Goto (2000)) and Reactome (Fabregat et al. (2017)). However, despite the enormous amount of available data, these databases are still incomplete and therefore have partial information.

In this case study, we have chosen *Basigin* (BSG), also known as CD147 or EMMPRIN, as a starting point to construct a protein-protein functional network. This gene represents an example of a biological element that should be supplemented to the KEGG network since it is not currently described in their pathways. BSG is a transmembrane glycoprotein of the immunoglobulin superfamily, expressed in many tissues and cells. It is known to participate in several highgly relevant biological and clinical processes. Furthermore, BSG is a crucial molecule in the pathogenesis of several human diseases (Xiong et al. (2014)).

Missing a crucial gene within a biological network can compromise scientists’ efforts to understand certain molecular mechanisms. However, the most reliable approach to date remains the manual curation through careful and time-consuming literature analysis. On the other hand, a manually constructed network provides partial information due to the limited number of articles that a scientist could read.

**FIGURE 8:**
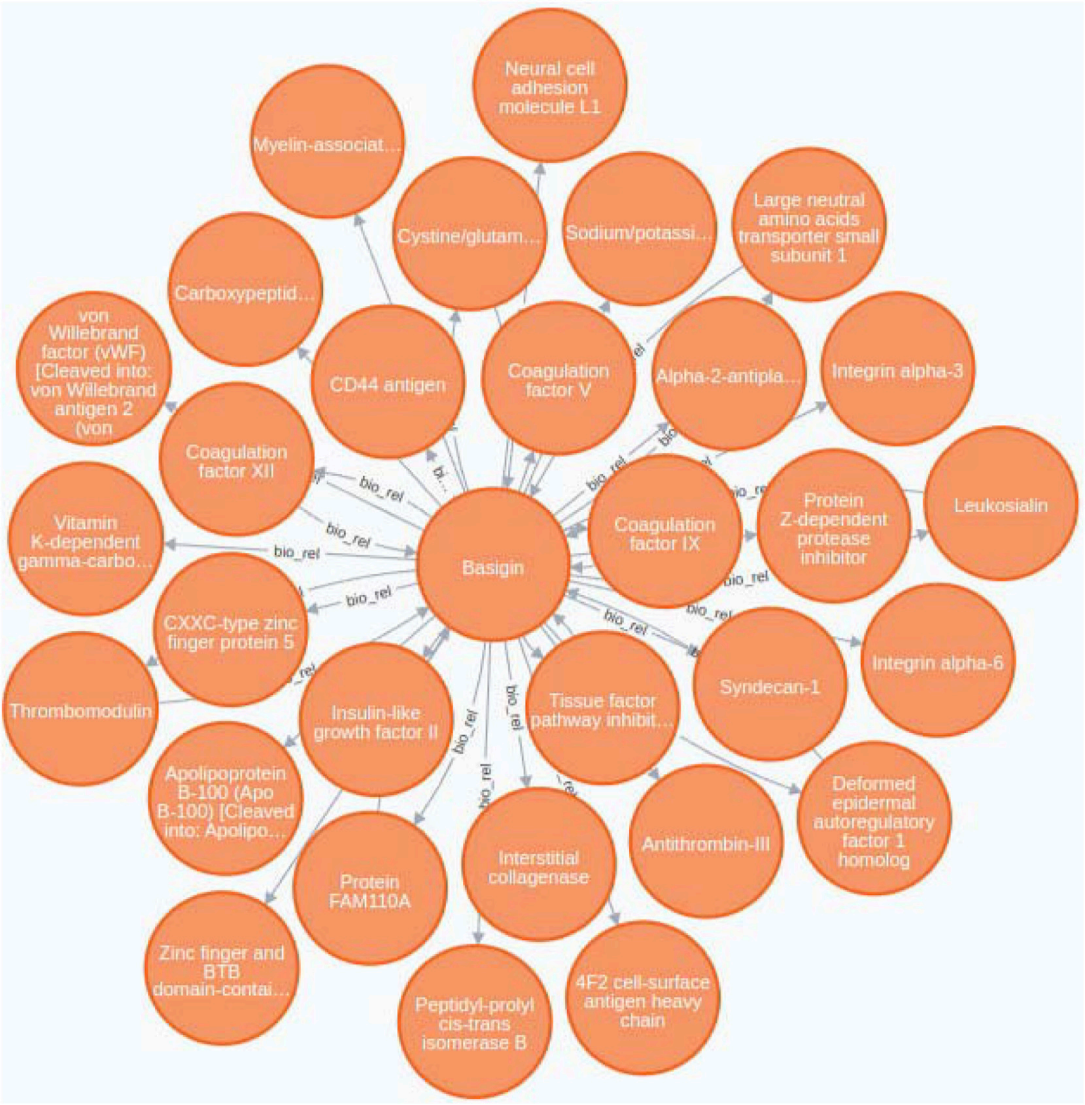
Basigin-Proteins interaction network. It has been created using the Neo4j user interface. In addition a limit of 30 nodes has been set. BioTagme and STRING edges have been merged in a single one.

Our case study tackle this issue by providing a practical example of how BioTagME can create valuable networks (Figure 8) by analyzing a large sets of PubMed abstracts. In addition, such a network has been compared with STRING to assess sensitivity and specificity.

Through BioTagMe, we inferred 426 true positive relations and 38 false negatives. Qualitatively, this network includes most of the interconnections mentioned in STRING, thus providing a reliable and comprehensive overview of the molecular function of *Basigin*. Quantitatively, BioTagME achieved a sensitivity of 91.8%, and a specificity of 94.8%.

### 3.2 Case Study 2

The second case study aims to build a general functional network related to the “blood coagulation pathway” and other biological entities (i.e. diseases, genes).

Blood coagulation is a complex chain process involving a series of stimulus responses in conjunction with coagulation factors and enzymes, whose intent is to stop blood fluxes when a vascular tissue injury occurs (Ngo et al. (2012)).

To evaluate the quality of BioTagME, our network (Figure 6) is compared with a “literature network” (generated by data and relationships into the external databases, Table 1) in terms of sensitivity and specificity.

BioTagMe was able to infer 54 true positive and 23 false negative. Quantitatively, We achieved a sensitivity of 70.12%, and a specificity of 96.43%. Indeed, we could predict the relation between blood coagulation and PROS1 (Figure 6). Such gene plays a crucial role on the mechanism of PtdSer exposure during immunity and blood coagulation (Wang et al. (2022)).

**FIGURE 9:**
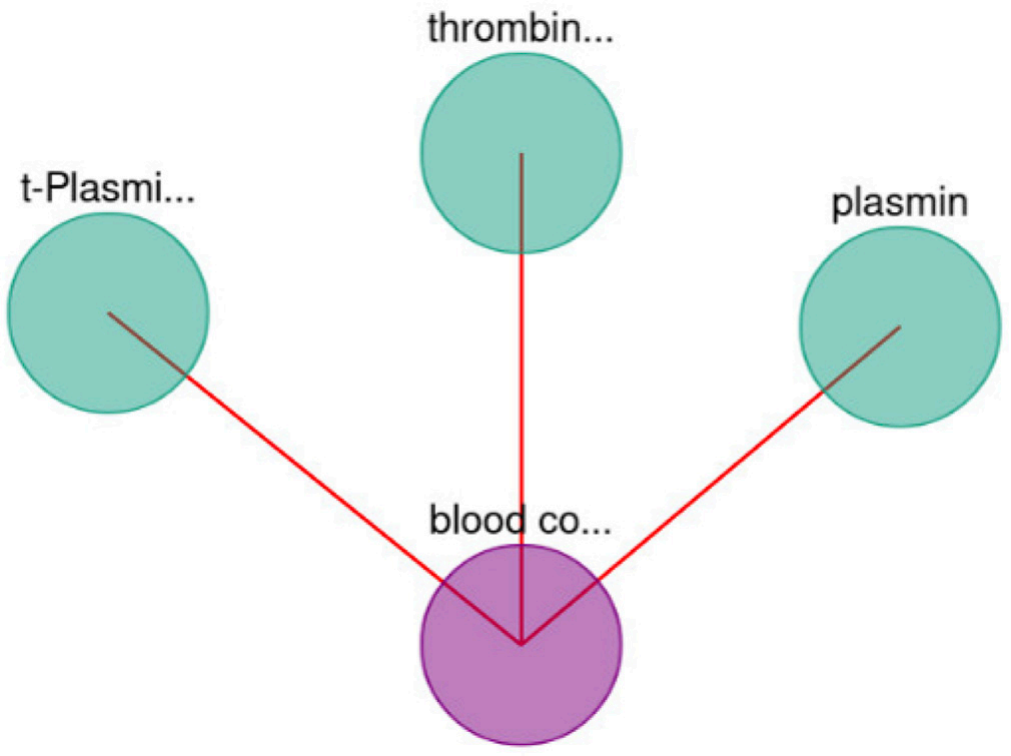
Blood coagulation and enzymes interaction.

Moreover, BioTagME could predict the relations among blood coagulation and the thrombin and plasmin enzymes (Figure 9). The role of Thrombin enzyme is to catalyze the initiation and propagation phases of blood coagulation. In addition, it converts soluble fibrinogen to insoluble fibrin (Becker et al. (2013)).

## 4 Conclusion

In this paper, we have implemented the BioTAGME framework for building offline biological knowledge graphs from all documents’ titles and abstracts in PubMed. First, the graph’s nodes (biological entities) have been extracted by TAGME. The edges, instead, have been predicted through the combination of the DT-Hybrid algorithm score and the TAGME relatedness computation. Such predicted edges have also been enriched with literature evidence resulting from laboratory experiments, biological, and biophysical processes (extracted from the connections among external databases), and protein-protein relationships in STRING. Moreover, an uploader module has been implemented to download and annotate new documents in PubMed to keep the graph up-to-date. Finally, the main pipeline (pipeline one) has been implemented using the Spark Framework to distribute the computation among several machines. Future works will include: 1) construction of knowledge-graphs based on open-access documents’ title, abstract and full-text in PubMed and PubMed Central; 2) implementation and integration of new prediction algorithms to improve and increase the prediction of the relationship among biological entities; 3) implement a TAGME version based on a biological Wikipedia corpus (no biological pages will be pruned); 4) development of a new search panel to enable advanced queries in the knowledge-graph. Such a panel will provide: algorithms for community detection (clustering); matching, shortest path, and k-shortest path based on BioTagME score, nodes and edges types, publication date, etc; centrality measures; cypher free text for writing custom queries. Moreover, we will add a list of sentences (where possible) to describe predicted relationships.

### 4.1 Permission to Reuse and Copyright

Figures, tables, and images will be published under a Creative Commons CC-BY license, and permission must be obtained for the use of copyrighted material from other sources (including re-published/adapted/modified/partial figures and images from the internet). It is the responsibility of the authors to acquire the licenses, follow any citation instructions requested by third-party rights holders, and cover any supplementary charges.

## Data Availability

The datasets presented in this study can be found in online repositories. The names of the repository/repositories and accession number(s) can be found in the article/Supplementary Material.

## Footnotes

1
http://string-db.org/.

2
https://neo4j.het.io/browser.

3
https://reactome.org.

4
https://lhncbc.nlm.nih.gov/ii/tools/SemRep_SemMedDB_SKR/SemRep.html.

5 if the url does not work, more information about a possible new url is reported within the readme of the repository: https://github.com/Anto188bas/biotagme_docker.git.

6
https://en.wikipedia.org/wiki/Portal:Biology.

7
https://tagme.d4science.org/tagme/.

8 Cypher is Neo4j′s query language to retrieve data from the graph, and was inspired by SQL.
